# Unveiling the potential of beetroot leaf as a sustainable source of proteins: insights into ultrasound-assisted extraction, functional properties and in vitro digestibility

**DOI:** 10.1016/j.ultsonch.2026.107751

**Published:** 2026-01-24

**Authors:** El Mehdi Raoui, Sofia Gruber, Milad Hadidi, Wisnu Arifan Anditya Sudjarwo, Alexander Einschütz Lopez, Jose L. Toca-herrera, Christian Leopold Lengauer, Marc Pignitter

**Affiliations:** a Institute of Physiological Chemistry, Faculty of Chemistry, University of Vienna 1090 Vienna, Austria; b Vienna Doctoral School in Chemistry (DoSChem), University of Vienna, Vienna, Austria; c Institute of Biophysics, Department of Bionanosciences (DBNS), University of Natural Resources and Life Sciences (BOKU), 1190 Vienna, Austria; d Institute of Mineralogy and Crystallography, Faculty of Earth Sciences, University of Vienna 1090 Vienna, Austria

**Keywords:** Ultrasound, Beetroot leaf protein, Sustainability, Functional properties, Plant proteins, Future food

## Abstract

Beetroot leaf, typically discarded as agricultural waste, is a promising source of plant-based proteins. With growing interest in sustainable and eco-friendly food production, extracting high-quality protein from such by-products supports a circular economy. This study optimized ultrasound-assisted alkaline extraction (UAAE) using a Box-Behnken design to maximize protein yield and content from beetroot leaves. The combination of ultrasound and alkaline treatment has been shown to enhance extraction efficiency and protein techno-functionality in comparison with conventional alkaline method (CAE). Optimal UAAE conditions (40 min, pH 11, 27.2 °C) yielded a protein content of 73.4% with a 10% extraction yield. Fourier transform infrared spectroscopy analysis confirmed that the secondary structure of beetroot leaf protein (BLP) obtained by UAAE remained intact, while microscopic analysis revealed a more compact globular morphology. Additionally, BLP obtained by UAAE showed better heat resistance and less aggregations, supported by a higher absolute zeta potential value (−31.06 mV, compared to −24.03 mV for BLP obtained by CAE). Both BLP obtained by UAAE and CAE displayed proportional increases in foaming capacity and stability with higher protein concentrations. UAAE led to improved digestibility of the BLP compared to legume protein isolates such as soy and pea protein. These findings highlight UAAE as an efficient method to produce high-quality protein from beetroot leaves, suitable for vegan foods, supplements, and pharmaceuticals.

## Highlights

• UAAE was optimized with Box-Behnken design for beetroot leaf proteins.
• Optimized UAAE yielded 73.4% protein content with 10% extraction yield.
• FTIR and microscopy confirmed intact structure and uniform globular proteins.
• UAAE improved heat resistance, reduced aggregation, and increased zeta potential.
• UAAE enhanced digestibility, supporting vegan food and supplement applications.

## Graphical abstract

**Figure:**
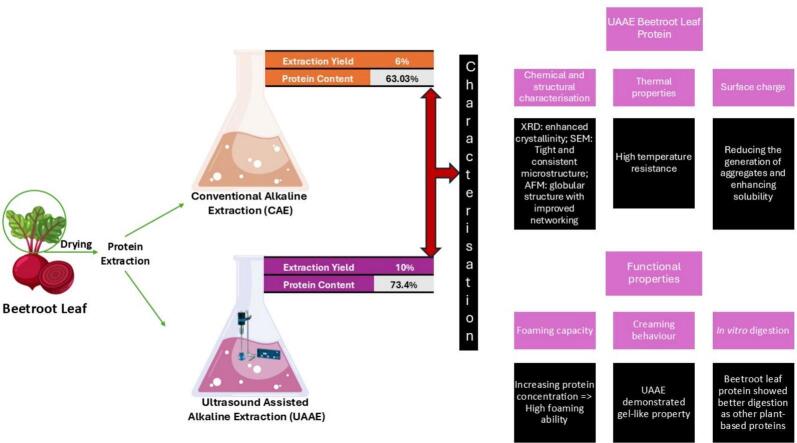

## 1 Introduction

The expanding global population presents a significant challenge to the accomplishment of a nutritious and sustainable diet. The ongoing search for sustainable sources to enhance food production and ensure future food security is a persistent challenge [1]. Protein is a fundamental component of human health, playing a crucial role in various biological processes including the formation of cells, tissues, enzymes, hormones, and antibodies [2], [3]. While animal-derived proteins are a source of all essential amino acids, they are also associated with significant environmental impacts, including high greenhouse gas emissions, pollution, and deforestation [4]. In contrast, plant-based proteins are more sustainable and cost-effective, although they may lack one or more essential amino acids. However, combining multiple sources can achieve a complete amino acid profile [5], [6]. In addition to their nutritional value, plant-based proteins have been demonstrated to contribute to the reduction of the risk of malnutrition, cardiovascular disease, diabetes, and certain cancers [7].

The increasing demand for sustainable protein alternatives has prompted interest in green leaf proteins (GLPs), which can be extracted from agricultural by-products such as leaves and stems. It is therefore important to note that these materials are rich in protein, vitamins, dietary fibre and essential fatty acids, yet are frequently discarded or used as low-value animal feed [8]. The valorisation of these residues is in alignment with the objectives of the circular bioeconomy and “zero-waste” strategies, as it involves the transformation of waste streams into valuable food ingredients [9].

Among GLP sources, the leaves of beetroot (*Beta vulgaris L. var. conditiva*) are particularly promising yet underexplored. Whilst the bulbs are widely utilised in the food industry for juice, pulp, and as a natural colourant (E162), the leaves represent up to 25–30% of total plant biomass and contain 25–35% crude protein on a dry-weight basis [10]. Studies on sugar beet and beetroot leaves have shown their high content of soluble proteins, such as Rubisco, as well as high antioxidant activity and mineral content [11], [12]. However, existing research has largely focused on the utilisation of sugar beet leaf for the purpose of pigment and fibre production rather than protein isolation. Furthermore, there is a lack of research that characterises beetroot leaf proteins (BLP). This gap emphasises the potential of beetroot leaves as an underutilised and sustainable protein source that is suitable for incorporation into plant-based food systems.

Alkaline extraction is considered to be one of the most widely utilised techniques for the extraction of proteins from plant materials, primarily due to its simplicity and cost-effectiveness [13], [14], [15]. In this process, the alkaline environment has been shown to facilitate the breakdown of plant cell walls and enhance protein solubility through increased electrostatic repulsion [16]. Despite these advantages, the conventional method often requires long processing times and considerable energy input, and it can partially denature proteins or compromise their functional properties [17]. In order to overcome these limitations, recent research has concentrated on integrating chemical and physical treatments with the aim of enhancing the efficiency and reducing the time required for the extraction process [18].

In this context, ultrasound-assisted alkaline extraction (UAAE) has gained attention as a promising alternative to conventional extraction methods. During the process, ultrasound waves generate cavitation bubbles that collapse and produce shear forces [19]. These forces are localised and effective in disrupting plant cell walls and enhancing mass transfer [20]. Consequently, protein solubility and overall extraction yield are significantly enhanced [21]. In comparison with conventional alkaline extraction (CAE), UAAE has been demonstrated to offer a number of distinct advantages, including reduced processing times, diminished energy requirements, and enhanced preservation of protein structure and functionality [22]. Nevertheless, the technique has not yet been systematically optimised for beetroot leaves, and its feasibility and effects on protein quality remain largely unexplored.

**Fig. 1:**
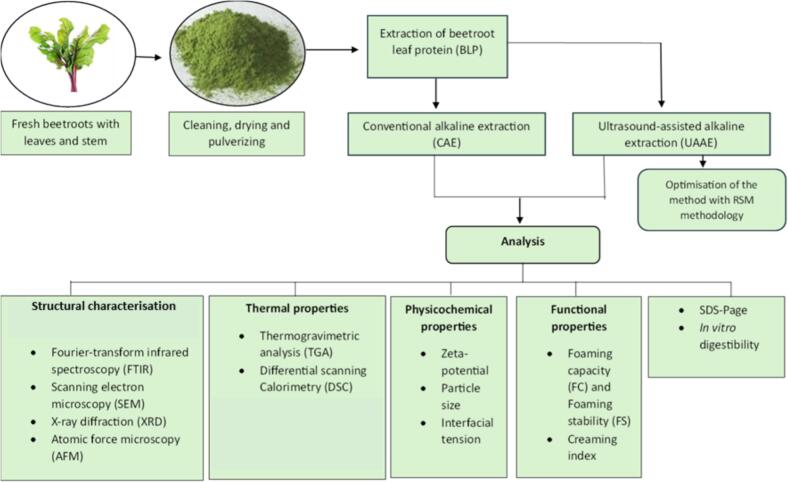
Scheme of the extraction process of protein from beetroot leaves with stems and the analyses for the structural characterisation, thermal, physicochemical, and the functional properties as well as in vitro digestion.

Therefore, the objective of this study was to extract and characterise proteins from beetroot leaves using two different methodologies, namely CAE and UAAE. The specific aims of the study were to: (i) optimize the UAAE conditions using response surface methodology, (ii) compare the structural and functional characteristics of beetroot leaf proteins (BLP) obtained by the two methods, and (iii) assess the in vitro digestibility of the extracted proteins (Fig. 1). Overall, this work introduces beetroot leaves as an underutilized yet promising source of sustainable plant protein and highlights the potential of ultrasound-assisted extraction to improve both the efficiency and quality of protein from beetroot leaf.

## 2 Material and methods

### 2.1 Raw materials and reagents

Fresh beetroots (*Beta vulgaris L. var. conditiva*) with leaves and stems were purchased from the local market in Vienna, Austria and dried, pulverized and stored at room temperature. The chemicals 2-mercaptoethanol (≥ 99%), acetone (≥ 99.8%), ammonium peroxydisulfate (≥ 98%), boric acid, brilliant Blue G 250, bromophenol blue, glycerol, glycine, hydrochloric acid (37%), isopropanol, Kjeldahl tablets, N, Ń–bis–methylene–acrylamide (99%), sodium chloride (≥ 99%), sodium hydrogen phosphate (≥ 99%), sodium hydroxide (32%), sodium hydroxide, p.a., ISO (≥ 99%), potassium chloride (≥ 99%), potassium dihydrogen phosphate (≥ 99%), sodium dodecyl sulfate (≥ 99.3%), sulfuric acid (≥ 98%), Tashiro indicator, tetramethylethylenediamine, tris(hydroxymethyl)aminomethane base (99%) were purchased from Carl Roth (Karlsruhe, Germany). 3–Aminopropyltriethoxysilane, acetic acid (≥ 99%) and acetonitrile (≥ 99.5%) were purchased from VWR (Radnor, USA). Acrylamide (≥ 99%), activated charcoal, methanol (≥ 99.9%), phthaldialdehyde (≥ 97%), protease Type I from bovine pancreas, sodium phosphate monobasic (≥ 99%), trypsin from porcine pancreas (Type II-S), and α – chymotrypsin from bovine pancreas (Type II) were purchased from Sigma-Aldrich (Darmstadt, Germany) and dimethylformamide, di–sodium tetraborate-decahydrate (99%) from Merck (Darmstadt, Germany).

### 2.2 Sample preparation

Fresh beetroot leaves with stems were initially washed with water to remove any accumulated dirt and then placed for drying at room temperature on paper towels for a period of two days. In order to ensure complete desiccation of the leaves with stems, they were placed on baking trays and subjected to an oven temperature of 50 °C for a period of 24 h. Furthermore, the dried beetroot leaves and stems were then ground using a Büchi Mixer B-400 (Büchi, Switzerland) equipped with two rotating knives, operating at 9,000 rpm and a motor power of 1,900 W, for approximately 1 min. The resultant powder was found to be of a uniform green leaf powder, with a particle size range of 150–300 µm, thus ensuring consistent material suitable for efficient solvent permeation during protein extraction.

### 2.3 Conventional alkaline extraction (CAE) of beetroot leaves protein

The beetroot powder was suspended in distilled water at a ratio of 1:20 g/mL, the pH of the solution was adjusted to 9 with 1 M NaOH at 25 °C, and continuously mixed at 200 rpm for 2 h. Then, the solution was centrifuged (Beckman Coulter Avant J-E, Krefeld, Germany) at 8000 rpm at 4 °C for 20 min to separate the supernatant from the precipitant, following the filtration of the supernatant using a vacuum pump (230 V, Edwards, (Dresden, Germany)). Moreover, the pH of the supernatant was adjusted to 4.2 with 1 M HCl and stored at 4 °C for 20 min [15]. This caused a change in the viscosity of the solution and the protein precipitate sank to the bottom. Afterwards, the suspension was centrifuged at 4000 rpm and 4 °C for 15 min. The resulting precipitate was washed with cold acetone to remove pigments, lipids, and other non-protein components, facilitating dehydration and improving protein purity, and then centrifuged at 4000 rpm and 4 °C for 10 min. All centrifugation steps were carried out using a refrigerated centrifuge maintained at 4 °C to prevent thermal denaturation. The pH of the aqueous precipitate was adjusted to 7 with 1 M NaOH and dried in the freeze-dryer [13].

### 2.4 Ultrasound-assisted alkaline extraction (UAAE) of beetroot leaf proteins

For the UAAE, the experiments were designed with Box-Behnken Design of the response surface methodology using the Design expert software version 12.0. Three independent variables, pH, sonication time and temperature, were selected with three different levels. The choice of pH, temperature, and sonication time was based on the fact that these parameters primarily impact protein solubility/dissociation (pH), the cavitation regime, mass transfer, and denaturation risk (temperature), and the total acoustic dose (time). Beetroot leaf powder was suspended in distilled water at a solid–liquid ratio of 1:20 g/mL. The pH was adjusted to 9, 10, or 11 with 1 M NaOH, and the suspensions were equilibrated to 25, 35, or 45 °C before sonication with a probe processor (SONOPULS HD 200, Bandelin electronic, Berlin, Germany) fitted with an MS 73 sonotrode. The instrument preset was fixed at amplitude/power MS 73/0 (for small volumes) with duty cycle = 10 (pulse mode) for all runs. The purpose of this was to ensure thermal stability, limited foaming, and safe cavitation in a small volume leaf slurry, with sonication times of 20, 30, or 40 min. The sonotrode was centered and immersed to a constant depth in a glass beaker, and the slurry was gently stirred to maintain homogeneity throughout treatment. Subsequently, the suspension was centrifuged at 8000 rpm and 4 °C for 20 min to separate the precipitate from the supernatant. The pH of the filtered supernatant was adjusted to pH 4.2 with 1 M HCl and stored at 4 °C. The supernatant and precipitate were separated by centrifugation at 4000 rpm and 4 °C for 15 min. Afterwards the precipitate was washed with 30 mL acetone and centrifuged again at 4000 rpm and 4 °C for 10 min. This step was of significant importance, as it facilitated the removal of colour, lipids, and other non-protein components. This process not only removed these impurities but also enabled the dehydration process and enhanced the purity of the protein. Finally, the protein precipitate was redispersed in 50 mL distilled water, neutralised to pH 7 with 1 M NaOH, and freeze-dried.

### 2.5 Structural characterization

#### 2.5.1 Fourier-transform infrared spectroscopy (FTIR)

Approximately 10–20 mg of BLP powder was compressed into pellets under a 1-ton load for structural analysis by FTIR spectroscopy. Spectra were collected at room temperature on an ALPHA FTIR spectrometer (Bruker, Ettlingen, Germany) operated in attenuated total reflectance (ATR) mode to allow direct comparison of proteins obtained by the different extraction routes. Data were recorded over 400–4000 cm^−1^, averaging 64 scans at a 4 cm^−1^ resolution. Because no colour differences were observed between CAE and UAAE-derived samples, ATR mode was appropriate [15]. Secondary structure was evaluated from the Amide I band (1600–1700 cm^−1^), which is highly sensitive to protein conformation [23], [24], [25]. Overlapping components in this region were deconvoluted in OriginPro 2024b, and the area of each peak was expressed as a percentage to estimate the corresponding secondary-structure elements. The following formula was used:

$$\mathrm{Area}=A\times \sigma \times \sqrt{2\pi }$$

$$\mathrm{Percentageofstructure}=\frac{\mathrm{Areaofthestructure}}{\mathrm{TotalAreaofAmideIBand}}\times 100$$

A is the peak amplitude and σ is the width of the peak.

#### 2.5.2 X-ray diffraction (XRD)

Assessing crystallite size and conformational changes is essential for understanding the thermal behaviour of proteins [26], [15]. Protein powders obtained by CAE and UAAE were gently packed into stainless-steel sample holders, and XRD patterns were collected on a Bruker D8 ADVANCE diffractometer (Bruker, Karlsruhe, Germany) in Bragg-Brentano geometry using Cu Kα radiation (λ = 0.15406 nm) and a LynxEye XE-T detector, at 40 kV accelerating voltage and 25 mA tube current. Data were acquired over 2θ = 2–65° with a step size of ≈0.1° and a scan speed of 1° s^−1^. Apparent crystallite size (D) was estimated with the Scherrer equation:

D=kλ/βcosθ

where k = 0.9 is the shape factor, λ is the X-ray wavelength (0.15406 nm), β is the full width at half maximum (FWHM) of the selected reflection in radians (after correction for instrumental broadening), and θ is the Bragg angle.

#### 2.5.3 Scanning electron microscopy analysis (SEM)

To assess how CAE versus UAAE affected protein structural integrity, BLP suspensions were prepared by dispersing 20 mg of powder (from either CAE or UAAE) in 1 mL of distilled water. Aliquots were deposited onto glass substrates, allowed to air-dry, and sputter-coated with a 10 nm chromium/gold layer using a MED 020 coater (BAL-TEC, Liechtenstein) to enhance conductivity and image contrast [7]. Imaging was performed on an Apreo scanning electron microscope (Thermo Fisher Scientific, MA, USA) operated at 2.0 kV and 0.10 nA, using the T1/T2 in-lens detectors, at 6,500 × magnification.

#### 2.5.4 Atomic force microscope (AFM)

##### 2.5.4.1 AFM sample purification and preparation

BLP powders obtained by CAE and UAAE were first purified to minimize pigment and non-protein contaminants. Samples were washed repeatedly with cold acetone (−20 °C) and centrifuged (4000 rpm, 15 min, 4 °C) to aid decolorization and improve downstream protein purification. The washed extracts (50 mg) were dispersed in PBS (10 mL) and centrifuged (4000 rpm, 10 min, 25 °C) to remove insoluble material. Subsequently, 5 mL of the clarified BLP solution was mixed with 10 mL PBS and transferred to a centrifugal filter unit (Vivaspin™ 20, Sartorius, Vienna, Austria), then centrifuged at 4000 rpm, 25 °C for 35 min.

##### 2.5.4.2 Substrate cleaning and functionalisation

Freshly cleaved mica discs (circular, 2.4 cm diameter) were cleaned by oxygen plasma (Brand Diener; 50 W, 60 s). Surfaces were then aminated by immersion in 1% (v/v) 3-aminopropyltriethoxysilane (APTES) in dry toluene for 1 h, rinsed thoroughly with N,N-dimethylformamide (DMF) and deionised water, and dried under a nitrogen stream.

##### 2.5.4.3 Protein immobilisation

For covalent attachment, 10 µL of the purified BLP solution (CAE or UAAE) was mixed with 200 µL 1-ethyl-3-(3-dimethylaminopropyl)carbodiimide (EDC, 100 mM) and 200 µL N-hydroxysuccinimide (NHS, 200 mM) in PBS for 30 min. The reaction mixture was then diluted to 10 mL with PBS (pH 7.2) and applied to the APTES-modified mica overnight at 4 °C. After incubation, substrates were rinsed with PBS and dried under nitrogen.

##### 2.5.4.4 AFM imaging and analysis

Topography was recorded in tapping mode (air) using a triangular silicon nitride cantilever (DNP-S10, Bruker; nominal spring constant 0.12 N m^−1^, tip radius ≈10 nm). Images were acquired at 512 × 512 pixels with a scan rate of 1–2 Hz. For each condition, three independent replicates were measured to assess repeatability. Images were processed and analysed using Gwyddion 2.62 and NanoScope Analysis 1.5.

### 2.6 SDS-Page

SDS-PAGE was carried out under reducing conditions using a 12% separating gel and a 5% stacking gel [7], [27]. BLPs obtained by CAE and UAAE were mixed with SDS-PAGE sample buffer (0.5 M Tris, 25% glycerol, 1.0% bromophenol blue, 10% SDS, 2-mercaptoethanol, and distilled water) and prepared at two final concentrations, 10% and 5% (w/v) to evaluate band visibility at different loadings. 15 μL of each BLP preparation and 15 μL of the protein ladder were loaded per lane. The running buffer was prepared as 10 × Tris-glycine-SDS (250 mM Tris, 1.92 M glycine, 1% SDS, pH 8.3) and diluted 1:10 to 1 × immediately before use (final: 25 mM Tris, 192 mM glycine, 0.1% SDS, pH 8.3). The PageRuler Plus Prestained ladder (Thermo Fisher Scientific, Schwerte, Germany; 10–250 kDa) contains dye-stained proteins in 62.5 mmol Tris-H_3_PO_4_ (pH 7.5, 25 °C), 1 mmol EDTA, 2% SDS, 10 mmol DTT, 1 mmol NaN_3_, and 33% glycerol. Electrophoresis was performed in a peQLab EV233 chamber at 100 V for 15 min, then 150 V for 60 min. Gels were released from the plates, the stacking gel was removed, and the resolving gel was stained in Coomassie Brilliant Blue solution (18% acetic acid, 0.5% Coomassie Brilliant Blue, 81% water) for 20 min. Destaining was carried out in 30 mL of destain (7.4% acetic acid, 7.4% methanol, 85.2% water) for 16 h with gentle agitation until a clear background was obtained. Gels were then stored in Milli-Q water at 4 °C overnight and subsequently placed on a glass plate for imaging; the BLP banding patterns were compared against the reference ladder to assess apparent molecular masses.

### 2.7 Amino acid composition

The amino acid profiles of BLP obtained by CAE and UAAE were quantified following the method described by Shen et al. (2020), with modifications. In order to hydrolyse the protein, 100 mg of CAE and UAAE proteins were added into 10 mL 6 N HCl and then incubated at 105 °C for 24 h. After that, samples were cooled down and diluted to 50 mL with 100% LCMS-grade water. Because strong acid/heat conditions destroy Tryptophan (Trp) and oxidize/degrade sulphur amino acids (Cys, Met), the following treatments have been applied, in which Trp was quantified from a separate alkaline hydrolysate (4 M NaOH, 110 °C, 18 h), followed by neutralisation, dilution and analysis. Cys and Met were stabilised by performic acid pre-oxidation (ice-cold formic acid:H_2_O_2_, 9:1 v/v, 0–4 °C, 60 min). In order to minimise the presence of artefacts, 0.1–1% phenol was incorporated into the acid hydrolysis process with the objective of protecting Tyr. Next, 2 mL of the diluted and hydrolysed samples were subjected to filtration via a 0.2  μm polytetrafluoroethylene (PTFE) filter prior the derivatisation step. Subsequently, amino acids were derivatised using 6-aminoquinolyl-N-hydroxysuccinimidyl carbamate (AQC). The derivatised amino acids were then detected using a UV Diode-Array Detector (DAD) SPD-M30A at a wavelength range of 200–400 nm. Standard curves were generated for each amino acid, with α-aminobutyric acid (AABA) serving as the internal standard.

The amino acid profile was determined using a Shimadzu HPLC system which was equipped with a SIL-20AC autosampler maintained at 20 °C and an LC-20AD binary pump. The column oven temperature was held steady at 37 °C, and separation was carried out on an AccQ-Tag reversed-phase column (4  μm, 3.9 × 150  mm; WAT052885, Waters, Milford, MA). The analysis employed a gradient elution with three different solvents: Eluent A (1:10 dilution of AccQ-Tag Eluent A concentrate in Milli-Q water), Eluent B (acetonitrile with 0.1% formic acid, HPLC grade), and Eluent C (100% water). The specific gradient conditions followed are provided in Table S1.

### 2.8 Thermal characteristics

#### 2.8.1 Thermogravimetric analysis (TGA) and Differential scanning calorimetry (DSC)

Both TGA and DSC analyses were conducted using a TGA/DSC system (Mettler Toledo GmbH, Vienna, Austria), under a constant nitrogen flow of 80 mL/min. The temperature was gradually increased from 25 °C to 650 °C at a rate of 10 °C per minute. During TGA, physical and chemical changes such as mass loss dependent on both temperature and time were observed. DSC, widely used in food science, was applied to study the kinetic and thermodynamic behaviour of proteins, particularly focusing on structural transitions triggered by temperature changes [7].

### 2.9 Interfacial physicochemical properties

#### 2.9.1 Zeta potential

The zeta potential of proteins isolated using CAE and UAAE was measured as described by Ortega et al. [15]. For this, a 1:2 mass-to-volume mixture of BLP and distilled water was prepared. Measurements were carried out using a Nano Zetasizer (Malvern, UK), which operates via electrophoretic light scattering and detects particle sizes ranging from 3.8 nm to 100 µm in diameter.

#### 2.9.2 Interfacial tension

The interfacial tension-reducing effect of the extracted BLP was measured at the rapeseed oil–water interface using the pendant-drop method with an interfacial tensiometer (Drop Shape Analyser DSA30, Krüss, Germany). Therefore, a suspension with 2.5 mg of BLP after CAE and UAAE and 30 mL of distilled water was prepared and put in a glass cell, while rapeseed oil was loaded into a syringe. In the glass cell, a droplet of maximum volume of 10 μL was formed at the inverted needle tip and immersed in aqueous solution in the glass cell. The densities of the test materials at 25 °C were as follows: canola oil (0.915  g/cm^3^), CAE proteins (1.090  g/cm^3^), and UAAE proteins (1.005  g/cm^3^). The droplet was monitored for 1800 s, with interfacial tension recorded every 10 s to capture the adsorption kinetics. The interfacial tension was measured as well as calculated using the software ADVANCE (Krüss, Germany).

### 2.10 Functional properties

#### 2.10.1 Creaming index

Oil-in-water emulsions were prepared using BLP isolated via CAE and UAAE at concentrations of 5%, 10%, and 15%. Each formulation was homogenized using a T18 digital ULTRA-TURRAX (IKA, Staufen, Germany) at 20,000 rpm for approximately 3 min. After homogenization, the emulsions were transferred into sample tubes and stored at both room temperature and 4 °C for one week. The tendency of phase separation was monitored, and the stability was calculated based on the creaming index using the following equation:

$$CI\left(\%\right)=\left(\frac{\mathrm{Hl}}{\mathrm{Ht}}\right)\times 100$$

Hl stands for the dimension of the bottom liquid layer and Ht for the denoted overall height.

#### 2.10.2 Foaming properties

The foaming capacity and the foaming stability were used to characterize the foaming properties [28]. For the following experiments, two different concentrations 10% and 15% of BLP after CAE and UAAE were prepared as triplicates. Furthermore, 15 mL of the BLP and distilled water suspension was homogenized at 20.000 rpm for 2 min using a T18 digital ULTRA-TURRAX (IKA, Staufen, Germany). For the calculation of foaming capacity (FC) and stability (FS), the following equations were used:

$$FC\left[\%\right]=\left[\frac{V1}{V}\right]\times 100$$

$$FS\left[\%\right]=\left[\frac{V2}{V1}\right]\times 100$$

V1 stands for the volume immediately after whipping, V is the total volume before homogenization, V2 stands for the volume after 10 min and 1 h.

### 2.11 *In vitro* digestion

For the in vitro protein digestibility (IVPD) the multi-enzyme method from Ortega et al., [15] was used. Suspension with BLP after the CAE and UAAE were prepared with a protein concentration of 5.4 mg/mL in 50 mL of distilled water. In addition, the suspensions were incubated in a water bath at 37 °C for about an hour. For the digestion three enzymes were used: trypsin from pork pancreas Type II-S (1.6 mg, specific activity: 1,000 – 2,000 N-Benzoyl-L-arginine ethyl ester units/mg solid), α-chymotrypsin from bovine pancreas Type II (3.1 mg, specific activity: ≥ 40 units/ mg protein) and protease Type I (1.3 mg, specific activity: ≥ 5 units/mg protein), which were added to the protein suspension. The pH was measured using pH-meter (pH 1100H, phenomenal ®, VWR, Germany) at the beginning of the digestion. The suspension was incubated for another 10 min in a water bath at 37 °C. Afterwards, the pH of the sample with the enzyme solutions was measured. For the calculation of IVPD, the following formula was used:

IVPD=65.66+18.1ΔpH_10min_

### 2.12 Statistical analysis

All experiments were conducted at least three times with freshly prepared samples, and measurements were performed in triplicate. Statistical analyses and visualisations were performed in GraphPad Prism 10.2.3 (GraphPad Software, San Diego, CA, USA), and results were reported as mean ± SEM. Experimental data were analysed by one-way analysis of variance (ANOVA) followed by Tukey’s multiple-comparison test, with statistical significance set at p < 0.05. Unpaired t-tests were used to compare CAE and UAAE for extraction yield, Kjeldahl results, in vitro digestion, and XRD analyses. Moreover, the software OriginPro 2024b was used for analysing and visualising the secondary structure of BLP after CAE and UAAE.

#### 2.12.1 Response surface methodology (RSM)

RSM was employed as a statistical approach to optimise the extraction process, specifically aiming to maximise both the protein content (%) and extraction yield (%) from beetroot leaves. The experimental design included three key independent variables: sonication time (20, 30, and 40 min), pH levels (9, 10, and 11), and extraction temperature (25 °C, 35 °C, and 45 °C). Each variable was tested at three coded levels: (−1), (0), and (+1). Additionally, four replicates at the central point were included to improve the accuracy of the model, resulting in a total of 16 experimental runs. These parameter ranges were selected based on previously published research[13], [29], [15]. The data collected from these experiments were analysed using Design Expert version 12.0, with model fitting and validation performed through analysis of variance (ANOVA) using a quadratic model. This optimisation process allowed for the identification of the most effective conditions for extracting protein from beetroot leaves and stems.

## 3 Results and discussion

### 3.1 Effect of different factors on the responses

Table 1 summarised the extraction yield and protein content obtained in the 16 UAAE runs, while Table 2 has reported the ANOVA for the quadratic model. The model demonstrated a strong fit to the data, as indicated by the non-significant lack-of-fit and the high coefficients of determination (R^2^ = 0.9157 for extraction yield; R^2^ = 0.9808 for protein content). These findings suggest a robust correlation between the predicted and observed values, and successful optimisation of BLP extraction [30], [15]. Comparable adequacy metrics (high R^2^ with non-significant lack-of-fit) have been reported for ultrasound-assisted, RSM-guided protein isolation in other matrices, including Dolichos lablab protein and faba bean isolates, supporting the robustness of the present model form [31], [32]. Consistent with predictions, the adjusted R^2^ values exhibited a minor decline yet remained closely aligned with R^2^ (0.7892 for yield; 0.9520 for protein), thereby demonstrating robust predictive accuracy with minimal error in estimation. The coefficient of variation (CV) further elucidated the precision of the model, with yield exhibiting a CV of 12.92% and protein content showing a CV of 3.05%. In general, a CV of approximately 10% is considered to represent a satisfactory correlation between observed and predicted values [15]. Consequently, the observed dispersion around the model was found to be minimal for yield and negligible for protein. In combination with the F- and p-values presented in Table 2, these diagnostics verify the statistical significance and reliability of the modelled responses (F = 7.40 for yield; F = 34.07 for protein). The Adeq Precision values for both responses exceeded 4, thereby confirming an adequate signal-to-noise ratio for navigating the design parameters [33], [34]. Collectively, these indicators (R^2^/adj. R^2^, CV, non-significant lack-of-fit, F-values, and Adeq Precision) demonstrate that the model is reliable for both optimisation and prediction [30], [15], (Olalere & Gan, 2023).

**Table 1:** Design of the experiments performed, and results obtained from the optimization process.

Run	Factors				Responses	
	A	B	C			
	Sonication time (min)	pH	Temperature [°C]		Extraction yield [%]	Protein content [%]
1	20 (−1)	9 (−1)	35 (0)		7.1	72.50
2	40 (+1)	10 (0)	25 (−1)		7.3	69.76
3	20 (−1)	11 (+1)	35 (0)		6	75.38
4	30 (0)	10 (0)	35 (0)		6.5	58.20
5	30 (0)	9 (−1)	25 (−1)		7.1	53.75
6	20 (−1)	10 (0)	25 (−1)		7	66.27
7	30 (0)	10 (0)	35 (0)		5.5	55.31
8	40 (+1)	10 (0)	45 (+1)		3.1	53.41
9	30 (0)	11 (+1)	45 (+1)		3.5	57.37
10	30 (0)	11 (+1)	25 (−1)		9.7	56.87
11	30 (0)	9 (−1)	45 (+1)		5.5	45.96
12	40 (+1)	11 (+1)	35 (0)		6.8	72.01
13	30 (0)	10 (0)	35 (0)		5	60.98
14	40 (+1)	9 (−1)	35 (0)		3.6	64.34
15	30 (0)	10 (0)	35 (0)		5.8	58.16
16	20 (−1)	10 (0)	45 (+1)		7.4	73.27

**Table 2:** ANOVA of the quadratic model from the results of the Box-Behnken design of the response surface methodology.

		Extraction yield (%)			Protein content (%)	
Source	Coefficient	F-value	p-value	Coefficient	F-value	p-value
Model		7.24	0.0128		34.07	0.0002
A–Sonication time	−0.8375	9.17	0.0232	−3.49	27.09	0.0020
B–pH	0.3375	1.49	0.2682	3.13	21.88	0.0034
C–Temperature	−1.45	27.48	0.0019	−2.08	9.63	0.0210
AB	1.07	7.55	0.0334	1.20	1.60	0.2532
AC	−1.15	8.64	0.0260	−5.84	37.98	0.0008
BC	−1.15	8.64	0.0260	2.07	4.79	0.0713
A^2^	−0.0375	0.0092	0.9268	12.54	175.32	<0.0001
B^2^	0.2125	0.2951	0.6065	0.3506	0.1369	0.7240
C^2^	0.5375	1.89	0.2185	−5.03	28.16	0.0018
Lack of fit		1.16	0.2774		0.3399	0.8005
C.V. %	12.92			3.05		
R^2^	0.9157			0.9808		
Adj-R^2^	0.7892			0.9520		
Adec precision	10.7113			20.2142		

The factor significance was response-dependent. For the extraction yield, sonication time and temperature were found to be significant (p ≤ 0.05), whereas pH was not. In contrast, the analysis of variance revealed that pH, sonication time, and temperature were all significant factors (p ≤ 0.05) (Table 2). Practically, this pattern suggests that mass-transfer-related kinetics (time/temperature) primarily impact recovery, while protein content is additionally sensitive to changes in solution chemistry (pH). The strength of the fit statistics and the factor-effect pattern are consistent with response-surface optimisations reported for plant matrices in related studies[30]. Furthermore, these findings align with the general guidance on model adequacy and interpretation of ANOVA metrics in DOE-based extractions (Olalere & Gan, 2023).

**Fig. 2:**
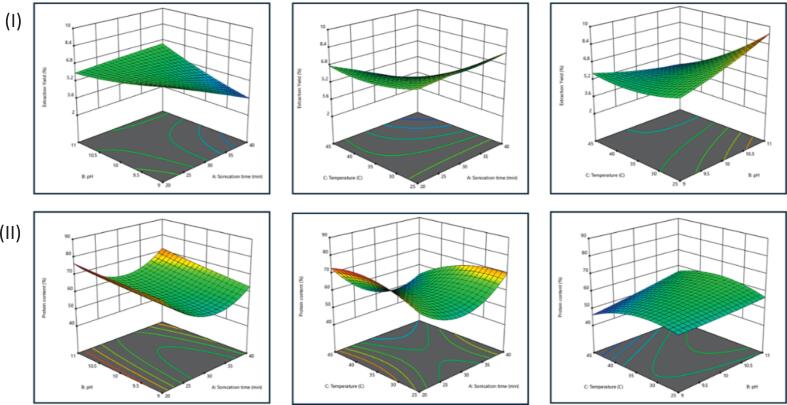
3D interaction between single factors (sonication time (A), pH (B) and temperature (C)) affecting extraction yield (I) and protein content (II).

In both responses, sonication time (A) and temperature (C) exerted a significant effect on the outcomes, while pH (B) demonstrated a response-specific role. For extraction yield, higher pH (B) combined with longer sonication time (A) increased recovery, and a similar improvement was observed for high pH (B) at room temperature (C). By contrast, elevated temperature (C) reduced yield (Fig. 2), consistent with protein aggregation and loss of solubility at higher thermal inputs [35], (Zakki et al., 2024).

With regard to the protein content, it was demonstrated that an elevated pH (B) and an extended sonication time (A) resulted in elevated values. This finding suggests that the combination of alkaline conditions (enhanced solubility above the isoelectric point) with ultrasound-induced cavitation enhances cell-wall disruption and protein release [35]. This phenomenon aligned with the established literature on the role of ultrasound in accelerating mass transfer, while pH is known to impact protein solubilisation and precipitation behavior [36]. Furthermore, the thermal energy generated during cavitation has been shown to influence protein conformation, which may help to explain the lower protein content observed at low pH (B) and high temperature (C) due to possible denaturation (Fig. 2) [37], [38]. In general, an increase in sonication time (A), room temperature (C), and elevated pH (B) resulted in enhanced extraction yield and protein content. In contrast, elevated temperature (C) led to a reduction in yield, likely attributable to aggregation and partial structural breakdown [39]. The response-surface equation for extraction yield is given in the following equation:

$$Y\left(EY\right)=5.70-0.8375A+0.3375B-1.45C+1.07AB-1.15AC-1.15BC-0.0375A^{2}+0.2125B^{2}+0.5375C^{2}$$

In consideration of the interaction between the three independent variables, the extraction yield was found to be influenced by the interaction between sonication time and pH (AB), sonication time and temperature (AC), and pH and temperature (BC). With regard to the protein content, it was found that the interaction between sonication time and temperature (AC) was the only factor that exerted an influence on the response. The interaction of sonication time and pH (AB) as well as pH and temperature had no effect on the BLP. The protein content was determined using the following equation:

$$Y\left(PC\right)=58.16-3.49A+3.13B-2.08C+1.20AB-5.84AC+2.07BC+12.54A^{2}+0.3506B^{2}-5.03C^{2}$$

### 3.2 Comparison of optimized UAAE with CAE

Using the 16-run dataset, the optimal UAAE conditions for beetroot-leaf protein extraction were pH 11, 40 min sonication, and 27.2 °C. In the experimental design, the UAAE model exhibited higher efficiency than the CAE, with an augmented extraction yield from 5.5 ± 0.5% (CAE) to 9.0 ± 1.12% (UAAE) and an elevated protein content from 57.42 ± 5.5% to 70.81 ± 1.57%. The protein content of dried beetroot leaves (22.04%) was consistent with previously reported values, thereby supporting the hypothesis that this by-product is a suitable source of protein [14], [27]. According to Balballi & Karabulut [40], the mechanism of CAE is based on the pH-driven solubilisation of proteins, yet the disruption of plant cells remains limited, thereby reducing the release of these proteins. In contrast, the UAE process incorporates acoustic cavitation, which has been shown to disrupt cell walls, enlarge the effective contact area between tissue and solvent, and accelerate mass transfer [8]. These mechanisms collectively contributed to the enhanced extraction and purity observed in this study. In practice, the ultrasound step accelerates the process and enhances energy efficiency, while preserving functionality when operated at moderate temperatures Chemat et al. [19]. Similar advantages of ultrasound-alkaline extraction have been reported for olive leaves (higher solubility and better foaming and thermal behaviour than with conventional extraction) [15], mulberry leaves (kinetic and thermodynamic gains with ultrasound-assisted cellulase) [41], and alfalfa leaves (ultrasound-, ultrafiltration- and alkaline precipitation-assisted processes, optimised by BBD/RSM) [13]. These findings are consistent with broader reviews of ultrasound-assisted extraction, which highlight cavitation-induced cell disruption, enhanced mass transfer and reduced processing times [42], [43].

### 3.3 Structural characterization

#### 3.3.1 FTIR analysis

**Fig. 3:**
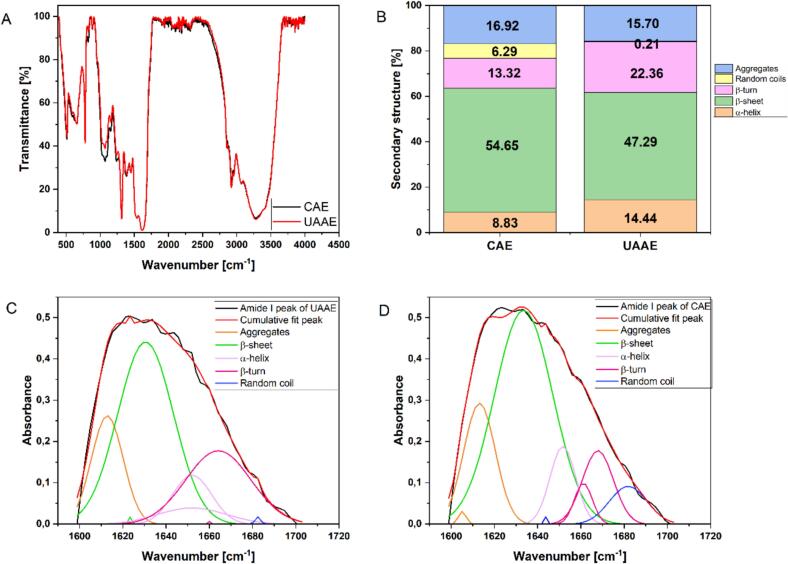
(A) FTIR-spectra of BLPs after CAE and UAAE; (B) Calculated secondary structure of BLPs after CAE and UAAE; (C) Deconvolution of amide I band into individual peaks of BLP after UAAE; (D) Deconvolution of amide I band into individual peaks of BLP after CAE.

Fourier-transform infrared spectroscopy (FTIR) is a widely utilised technique for the characterisation of protein functional groups, side chains, and secondary structures. As demonstrated in Fig. 3A, the spectra of BLP obtained by CAE and UAAE were broadly similar. The spectral region at 3100–3500 cm^−1^ corresponds primarily to N–H/O–H stretching of primary/secondary amines and amides, while the 2850–3000 cm^−1^ region reflects C–H stretching. The amide regions, amide I (1600–1700 cm^−1^), amide II (1550–1530 cm^−1^), and amide III (1260–1300 cm^−1^), are located within the range of 1700–1200 cm^−1^, as would be expected for proteins [44]. Only slight shifts were observed between BLP after CAE and BLP after UAAE, and amide-band positions changed minimally. This indicated that ultrasound under the applied conditions produced negligible changes in N–H bending and did not measurably disrupt the peptide backbone. In contrast, larger FTIR shifts have been reported for melon seed protein isolate and whey protein isolate under different processing conditionsYildiz [45], (Meng et al., 2021). These minor alterations suggested that UAAE did not cause significant changes to the primary structural elements of BLP, indicating that the protein matrix remained relatively stable. This observation is consistent with the findings of recent processing studies, which also reported that the backbone features of proteins remained comparatively stable under moderate ultrasound exposure [46]. Deconvolution of the amide I band (Gaussian fitting) resulted in the identification of peaks characteristic of α-helix (CAE: 1651.68 cm-1; UAAE: 1652.05 cm-1), β-sheet (CAE: 1633.53 cm^−1^; UAAE: 1623.36 and 1630.54 cm^−1^), β-turn (CAE: 1661.14 and 1667.88 cm^−1^; UAAE: 1660.05 and 1664.18 cm^−1^), random coil (CAE: 1643.72 and 1681.86 cm^−1^; UAAE: 1682.45 cm^−1^), and aggregates (CAE: 1605.14 and 1613.14 cm^−1^; UAAE: 1612.84 cm^−1^) (Fig. 3C and 3D). Quantitatively (Fig. 3B), UAAE-BLP exhibited slightly higher α-helix (14.44%) and β-turn (22.36%) and a lower β-sheet fraction (47.29%) relative to BLP after CAE, which demonstrated higher β-sheet (54.56%), aggregates (16.92%), and random coil (6.29%) (Fig. 3B). A previous study has demonstrated that a tendency towards β-structure is associated with reduced digestibility in certain plant proteinsLi et al. [47]. Meanwhile, increases in β-turn and random coil have been shown to correlate with enhanced enzymatic accessibility. It is important to note that the effect of ultrasound on the structure of the protein matrix can be dependent on the type of protein matrix. Studies of a mechanism have attributed such differences to cavitation-driven unfolding and slight disruption and rearrangement of disulfide linkages [48]. These can thereby rebalance secondary structure of proteins without large shifts in amide-band positions [49]. The findings demonstrated that ultrasound-alkali protocols have produced minimal secondary-structure changes in some matrices (e.g. evening primrose), consistent with the small FTIR shifts observed in the present study [29]. However, other systems show more pronounced responses depending on acoustic dose, pH, and temperatureYeasmen and Orsat [50]. The higher β-turn and lower β-sheet fractions in UAAE-BLP may suggest that the protein is more thermally stable, emulsifying and electrostatic, as has been observed for proteins with elevated β-structure content [51]. The findings of the present study indicated that, under moderate conditions, UAAE redistributes BLP secondary-structure elements to a slight tendency toward more α-helix/β-turn and less β-sheet, without significant backbone perturbation. This balance helps to elucidate how ultrasound-assisted extraction can enhance extraction efficiency and functionality while preserving structural stability, in keeping with findings that ultrasound can influence protein structure, interactions, and functionality [52], [53].

#### 3.3.2 X-ray diffraction (XRD) analysis

**Fig. 4:**
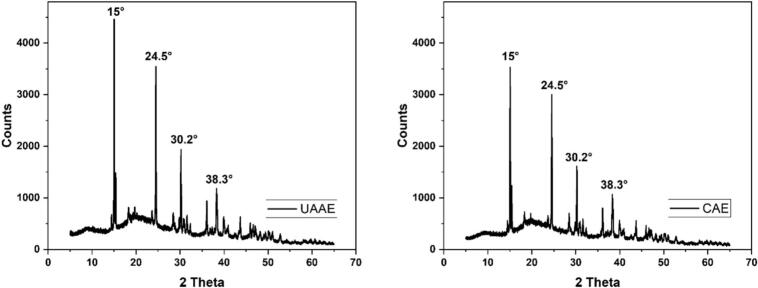
XRD-spectra of BLP after CAE and UAAE.

XRD was used to analyse the structure and crystalline phase of the isolates [54], and to determine intermolecular interactions among protein chains [55]. The diffraction angle (2θ) and peak intensity/width provide insight into crystallite size and ordering, and can reflect conformational rearrangements in protein assemblies [56]. The diffraction angle (2θ) and peak intensity/width provided insight into crystallite size and ordering, and can reflect conformational rearrangements in protein assemblies [57]. The BLP obtained by CAE and UAAE demonstrated four primary reflections at 15°, 24.4°, 30.2°, and 38.7° (Fig. 4). Despite the variation in absolute peak positions when compared to starch-rich systems, the overall pattern remains consistent with that observed in partially ordered biopolymer matrices. Analysis of rice protein-starch composites revealed reflections near 15.1°, 17.1°, 18.0°, 20.0°, and 23.0° [58], while rice starch demonstrated peaks at 15.9°, 18.8°, 23.3°, 27.4°, 32.1°, and 38.6° [59]. In the present study, BLP obtained by UAAE produced sharper, more intense peaks (narrower full width at half maximum) than BLP after CAE, together with a slight shift to lower angles, indicating higher crystalline ordering in BLP after UAAE (relative crystallinity 50.46 ± 25.6%) compared with BLP obtained by CAE (28.09 ± 3.11%). A similar increase in ordering has been reported for β-lactoglobulin-puerarin complexes after sonication [57] and for soy protein systems where ultrasound modified packing and interaction profiles, as detected by XRD [55]. Furthermore, ultrasonication has been demonstrated to enhance the packing of composite foods, thereby reducing their disorder. For instance, in potato powders fortified with protein, sonication has been shown to intensify specific reflections and modify crystallinity [60]. In contrast, protein isolates from sunflower meal exhibited a decrease in crystallite size following high-intensity ultrasound, accompanied by the formation of new bonds [61]. This emphasised that the structural outcomes are significantly influenced by the matrix, the acoustic dose, and the thermal exposure. Comparable ultrasound-linked crystallinity changes have been reported for faba-bean isolates optimised by RSM (XRD patterns of control vs ultrasound-treated protein) and for hybrid biopolymer systems where ultrasonication is used to induce ordering [31], [62]. Consequently, the more intense peaks and enhanced relative crystallinity observed in BLP obtained by UAAE indicate that cavitation induced localised changes in protein domain configuration during the extraction and drying processes, resulting in a more organised structure compared to that achieved by CAE. This interpretation is consistent with reports that acoustic fields can drive nucleation/re-ordering phenomena in soft matter and crystalline systems, with consequences for thermal stability and functionality (e.g., emulsification, water/oil binding) [60], [61], [63].

#### 3.3.3 Scanning electron microscopy (SEM)

**Fig. 5:**
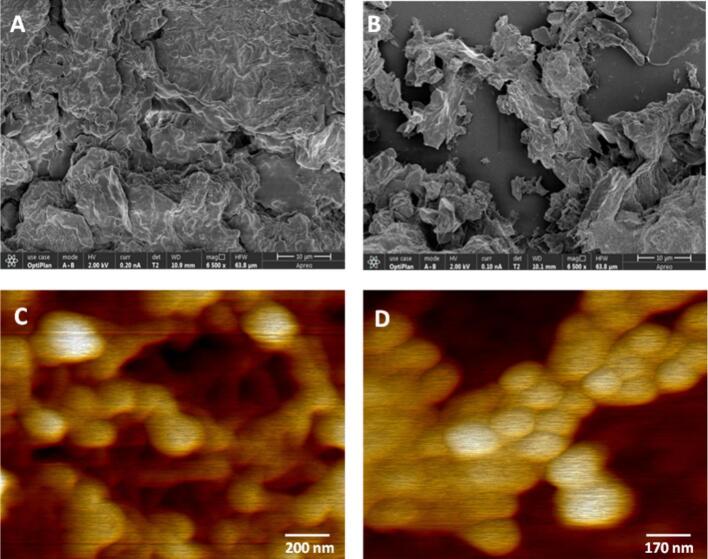
(A) SEM image BLP after CAE, (B) and BLP after UAAE, (C) AFM image BLP after UAAE, and (D) BLP after CAE.

Scanning electron microscopy is a standard tool for visualising protein microstructures and surface characteristics [64]. As demonstrated in Fig. 5A, the BLP obtained by UAAE exhibited a relatively smooth, fibre-like morphology, characterised by overlapping flakes of varying dimensions. The lamellar flakes indicate lateral association of protein aggregates, and the texture appeared dense but not granular. In contrast, BLP obtained by CAE (Fig. 5B) exhibited a coarse, angular surface with less coherent, cluster-like aggregates and more pronounced edges. These qualitative differences are consistent with ultrasound-driven changes in interparticle forces and dispersion, as indicated by controlled sonication increasing surface charge, exposing hydrophobic patches, and reducing particle size, thereby shifting aggregate structure and improving aqueous dispersibility [65], (Hu et al., 2013), [66]. Recent study on soybean protein similarly reported smaller particles, higher solubility, and more homogeneous micromorphology after ultrasound, supporting this interpretation [66]. The combination of alkaline pH and cavitation is suggested to have contributed to the UAAE surface features by enhancing cell-wall disruption and protein-solvent contact [67]. This is also supported by the finding from bioactive compounds from lotus leaf, where vacuum-ultrasound-assisted extraction modified recovery and microstructure [68], and from mulberry leaf protein, where ultrasound regulated conformation and aggregation via intermolecular force modification [69]. Similar microstructural improvements under ultrasound have been reported for sesame protein isolates (smoother, more continuous surfaces) and other plant proteins, frequently accompanied by gains in solubility, foaming or emulsification properties that depend on particle size, surface charge, and aggregate morphology. Consequently, the observations made through the use of SEM, which revealed the presence of smoother lamellae in BLP obtained by UAAE in comparison to the angular clusters observed in BLP obtained by CAE, are consistent with the findings reported in the literature. These reports indicated that moderate ultrasound can unfold proteins to a sufficient degree to disrupt compact clusters, decrease particle size, and enhance better dispersion without the complete denaturation of proteins [50].

#### 3.3.4 Atomic force microscopy (AFM)

AFM is a widely utilised instrument in the domain of food science, employed for the purpose of visualising protein topography at the nanometre scale [70]. As illustrated in Fig. 5C and 5D, the application of AFM has been employed to generate three-dimensional visualisations of BLP. Both images demonstrated the presence of globular structures despite exhibiting distinct organisational differences. In Fig. 5C, the globules revealed to be more interconnected, forming a less dense network with short fibrillar links between particles. Within Fig. 5D, the globules appeared to be more isolated and filamentous in nature, exhibiting a reduced number of junctions and a diminished apparent of network. This finding indicated that ultrasound-assisted extraction yields stronger interparticle interactions and more continuous assemblies, in contrast to the outcomes of conventional alkaline extraction, which generated less coherent clusters. Ultrasound has been demonstrated to induce changes through cavitation-induced shear [71]. These effects unfolded proteins, exposed hydrophobic regions and raised the zeta potential [72]. Furthermore, the effect of ultrasound cavitation led to changes in the protein structure, which resulted in a tightly arranged molecular network structures [73], [74]. Moreover, the cavitation effect of the ultrasound induced a transition in the protein morphology from fibril proteins to a tighter network structure, which was also visible in the extracted *Porphyra* yezoensis proteins [75]. The application of ultrasound in the extraction of mulberry leaf protein from plant-leaf matrices has been shown to enhance yield and modify microstructure [41]. This observation lends support to the theory that the combination of acoustic energy and alkaline conditions exhibited a dua-modality effect in the restructuring of protein assemblies. Thus, the nanostructure resulting from UAAE is characterised by enhanced connectivity and finer morphology, which is commonly associated with superior dispersion, elevated solubility, and augmented foaming/emulsifying properties. This is attributed to the fact that smaller, more uniform aggregates wet interfaces more efficiently and pack more compactly [76]. Soybean protein gel system demonstrated that ultrasound treatment of proteins results in emulsions with enhanced microstructure and stability, which is consistent with the observed trends [77].

### 3.4 Thermal characteristics

#### 3.4.1 Thermogravimetric analysis

**Fig. 6:**
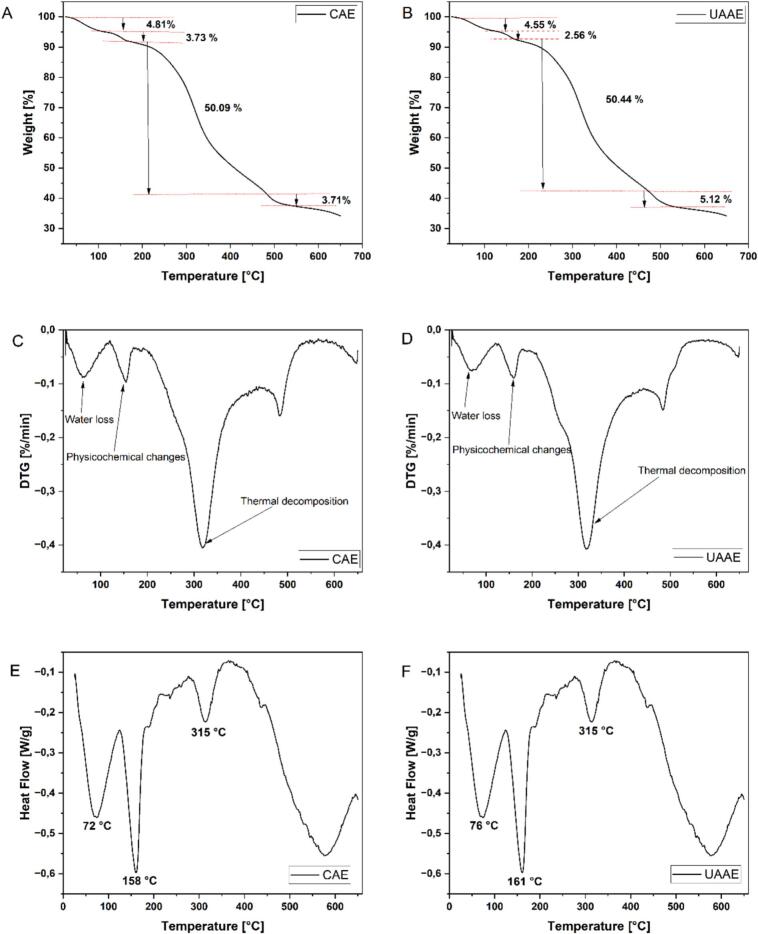
(A) TGA profile of BLP after CAE; (B) TGA profile of BLP after UAAE; (C), DTG of BLP after CAE; (D) DTG of BLP after UAAE; (E), and DSC of BLP after CAE; (F) DSC of BLP after UAAE.

Thermogravimetric analysis (TGA) was employed to monitor mass loss in BLP as temperature increased, enabling the primary stages of thermal denaturation to be elucidated [7], [78]. The two experiments (CAE and UAAE) exhibited four peaks in the derivative curves (DTG; Fig. 6C-D). The initial stage is characterised by the loss of adsorbed and bound water, with mass decreases of 4.81% for BLP obtained by CAE and 4.55% for BLP obtained by CAE (Fig. 6A-B). The second stage exhibited a reduced loss associated with preliminary structural reorganisation, including side-chain dehydration and the dissolution of weak non-covalent contacts, estimated at 3.73% (CAE) in comparison to 2.56% (UAAE). The lower second stage loss for BLP obtained by UAAE was consistent with the known effects of ultrasound-assisted extraction, where cavitation disperses aggregates and weakens hydrogen-bonding/hydrophobic associations without extensive degradation, often improving techno-functional properties [79], [21]. The third stage is characterised by the primary thermal decomposition of the protein matrix, which results in the most significant mass loss. It was found that BLP obtained by UAAE reached a maximum-loss peak of 50.44%, in comparison to 50.09% for BLP obtained by CAE. The BLP after UAAE trace exhibited a slight shoulder, which suggests a delayed decomposition process and, consequently, a more compact or organised network that is formed under ultrasound-alkali conditions. A similar matrix-dependent thermal response to ultrasound has been reported for pea, rice, and lupin proteins, with rice protein showing limited change in thermal parameters after ultrasound, emphasising that outcomes depend on composition and processing [80]. The fourth minor stage is likely to represent slow degradation of residual non-protein components (lignocellulosic material from beet leaves) not fully removed during extraction [15]. The TGA/DTG profiles indicated that UAAE maintains, and may slightly improve, thermal stability. This is consistent with cavitation-assisted rearrangement into a more cohesive molecular network. The processing advantages offered by UAAE are consistent with those already reported for ultrasound-assisted extraction from other plant sources.

#### 3.4.2 Differential scanning calorimetry (DSC)

DSC is a frequently employed technique in the field of food research, with the purpose of characterising the kinetic, structural transitions, and thermodynamic behaviour of proteins [7]. For the BLP, four endothermic transitions were observed for both CAE and UAAE derived samples, with differences in peak position and intensity. The instrument baseline placed the sample heat-flow below the reference, which is why the y-axis appears negative in Fig. 6E-F. It has been demonstrated that multiple transitions are consistent with heterogeneous protein domains and co-extracted constituents in plant leaf matrices. Nevertheless, single dominant transitions are often reported for more homogeneous plant proteins, such as those from *Amygdalus* pellucina pall seeds and fava beans [81], [82]. The initial two, relatively broad peaks exhibited divergent temperature patterns between CAE and UAAE, indicating early physicochemical rearrangements (side-chain dehydration, weakening of non-covalent interactions) that commence at discrete temperatures in the two preparations. The broadest points of the curve indicated that the transitions are occurring due to the overlap of multiple sub-transitions, rather than a single cooperative transition, which could be expected of a complex plant protein system. This observation is consistent with the results obtained from faba bean isolates [35]. The main transition (third peak) is indicative of the principal denaturation/decomposition phase. In BLP obtained by UAAE, this peak exhibited a slight shoulder relative to BLP obtained by CAE, indicating a slight delay in thermal breakdown and implying that a more cohesive network is formed under ultrasound-alkali conditions. Similar ultrasound-linked shifts in behaviour, such as onset and melting, have been reported for pumpkin-seed protein isolates when sonication time and power were modified [83]. The forth minor transition is likely indicative of the slow decomposition of residual non-protein material, such as lignocellulosic fragments from beet leaves, which is a common finding in leaf-protein preparations [15]. As demonstrated by the DSC profiles, ultrasound-assisted alkaline extraction has been shown to modify the thermal behaviour of BLP, with no indication of excessive degradation. The findings of this study demonstrate that the observed delay and modification of the primary transition in BLP obtained by UAAE are consistent with the cavitation-driven dispersion of aggregates and the partial rearrangement of intermolecular contacts. These effects have the potential to enhance process performance while preserving overall stability [84]. It has been demonstrated that matrix-dependent responses are to be expected, with rice protein showing limited thermal change after ultrasound, whereas pea and lupin systems can display measurable shifts in thermal events depending on acoustic dose and pH/temperature [80], [21]. Moreover, studies on olive-leaf, alfalfa-leaf and faba-bean proteins have reported that combining ultrasound with alkaline extraction or pH-shift can tune structure and functionality while maintaining thermal robustness [15], [13], [35], [79]. Additionally, these results are aligned with the findings reported from this study. UAAE has been shown to exhibit a promising balance between extraction and thermal resilience [85], which is advantageous for high temperature food processes where maintaining protein integrity and functionality is important [85].

### 3.5 Functional properties

#### 3.5.1 Creaming index

Three oil-in-water emulsions (5, 10, 15% w/w BLP) were prepared with proteins obtained by CAE and UAAE and stored for one week at 25 °C and 4 °C (Table 3). On the first day of the experiment, the protein concentration of both extracts had a significant effect on the creaming index, while storage temperature did not. After one week, 15% of BLP obtained by CAE differed significantly from 5% and 10% of BLP obtained by CAE, while 5% vs 10% of BLP after CAE did not demonstrate a significant difference, and once again, temperature had no detectable effect. For BLP obtained by UAAE, all three concentrations remained significantly different after a week, with no effect of storage temperature. Overall, the emulsions demonstrated a promising and temperature-insensitive creaming profile, with stability primarily influenced by protein concentration. This pattern corresponded to the findings reported in the literature concerning legume proteins, where elevated protein levels and the resultant decrease in droplet size/increase in interfacial surface area were found to influence gravitational stability to a greater effect than minimal fluctuations in storage temperature [86], [87]. Furthermore, mechanistic studies with pea and soy emulsions have shown similar behaviour, with concentration-dependent stability being linked to interfacial film formation, droplet size distributions, and continuous-phase viscosity [88], [89]. The enhanced concentration effect observed for BLP obtained by UAAE is consistent with ultrasound-related improvements in solubility, dispersion, and interfacial stability. High-frequency cavitation has been demonstrated to reduce aggregate size, increase surface charge, and expose interfacial areas. These effects are known to favour the formation of thinner, more cohesive interfacial films and lower creaming rates at a given protein concentration [90]. Recent studies have demonstrated that such ultrasound-enhanced gains in emulsion activity/stability indices have been reported for various plant proteins, including field bean, faba bean, and pistachio meal [91], [92], [93]. These findings are consistent with the broader existing literature on the use of ultrasound as a plant-protein modifier. The colour and appearance of the samples differed according to the extraction method employed. BLP obtained by CAE emulsions manifested as dark brown/grey at 4 °C and brown at 25 °C, whereas BLP obtained by UAAE emulsions were light grey at 4 °C and light brown/yellowish at 25 °C. The darker colours observed after CAE are consistent with the process of polyphenol-to-quinone oxidation under alkaline conditions. This process has been shown to promote covalent protein-phenol coupling and non-enzymatic browning [94]. Related quinone-mediated pathways have been extensively documented in the context of food systems [95]. It was observed that both groups of emulsions exhibited a gel-like texture during the storage period, suggesting a transition from a liquid to a gel state, driven by protein crosslinking (both physical and chemical processes) [96]. The gelation behaviour of plant proteins is understood to be affected by concentration, pH/ionic strength, and prior structuring (ultrasound) [97]. Protein-gel networks have been shown to enhance appearance, mouthfeel, and resistance to creaming under storage conditions [98]. Recent research on legume protein gels and plant-based emulsion gels demonstrated parallel mechanisms of network formation that stabilise emulsions without the use of animal gelatin [99]. These findings emphasised formulation mechanisms relevant to BLP. The results obtained in this study are consistent with the findings reported in the existing literature on the subject of ultrasound-modified plant proteins [100]. This included the concentration-driven creaming, the temperature-insensitive creaming, the lighter colour under UAAE, and the gel-like structuring. The application of ultrasound in extraction and treatment processes has been shown to enhance solubility, emulsion activity, and stability, often through the formation of smaller particles, higher zeta potential, and improved interfacial film properties. Moreover, a similar trend has been reported for faba bean and pea systems, where controlled sonication has been shown to shift both physicochemical and functional profiles in order to produce stable emulsions [35], [101].

**Table 3:** Creaming index of oil–water emulsion prepared with BLP obtained by conventional alkaline extraction and ultrasound-assisted alkaline extraction.

	Concentration [%]	CAE [%]	UAAE [%]
Day 1			
+25 °C	5 [%]	44.1 ± 1.31**^a^**	45.0 ± 2.04**^a^**
	10 [%]	36.3 ± 1.05**^b^**	11.1 ± 1.81^c^
	15 [%]	13.3 ± 1.18**^c^**	3.3 ± 1.18^d^
At +4 °C	5 [%]	44.2 ± 3.12**^a^**	40.0 ± 2.04**^a^**^, b^
	10 [%]	36.3 ± 2.09**^b^**	7.7 ± 2.72^c, d^
	15 [%]	12.5 ± 0.0**^c^**	7.4 ± 1.05^c, d^
Day 7			
+25 °C	5 [%]	48.9 ± 1.57**^a^**	38.3 ± 1.18**^b^**
	10 [%]	41.5 ± 1.05^a, b^	23.7 ± 3.78**^c^**
	15 [%]	19.2 ± 2.36**^c, d^**	12.5 ± 1.18^d^
At +4 °C	5 [%]	43.3 ± 5.14^a,^**^b^**	40.8 ± 1.18 ^a,^**^b^**
	10 [%]	40.0 ± 3.16^a, b^	24.0 ± 2.45**^c^**
	15 [%]	18.3 ± 1.18**^c, d^**	14.1 ± 1.05^d^

Note: Results are shown as mean ± SEM of three replicates. Significant differences (p < 0.05) within the same column are indicated by different letters (a, b).

#### 3.5.2 Foaming properties

Proteins exhibit a process of unfolding and redistribution during the whipping process, which facilitates their crosslinking at the air–water interface, leading to the formation of cohesive interfacial films [102]. As demonstrated in Table 4, the FC of BLP obtained by CAE and UAAE did not differ significantly at the same protein level, but increased with concentration (10% and 15% > 5%) for both extracts. The findings revealed that FC at 10% was elevated for both BLP after UAAE (133.0 ± 0.0) and BLP after CAE (130.67 ± 0.0), which exceeded the values reported for beet-leaf concentrates in Sedlar et al. [27] (FC ≈ 87.5 ± 1.32%; FS ≈ 48.6 ± 1.25%). This concentration dependence is consistent with the existing literature on plant proteins, which demonstrated that higher protein concentrations enhance interfacial surface, reduce bubble size, and strengthen the resulting films [103]. For foaming stability (FS), no significant differences were observed between extraction methods at 10% and 15%, and stability remained high over a period of one hour. The increase in protein primarily elevated the FC, while the FS remained constant. This finding is consistent with a scenario where the formation of a continuous viscoelastic film results in diminishing returns for protein, in terms of stability, within the measured timeframe [104]. It is important to note that BLP foams demonstrated a significant positive comparison with other green-leaf proteins [105]. Alfalfa and evening-primrose frequently exhibited higher FC but could be observed to have comparable or lower FS, while olive-leaf extracts generally exhibited lower FC and FS in comparison to BLP [29], [15]. Furthermore, comprehensive studies of leaf proteins (including Rubisco-rich isolates) have also revealed the presence of strong foaming properties, thereby affirming the viability of leaf fractions as effective foaming agents in the context of food products subjected to air exposure [106]. This finding is supported by the mechanistic results, which indicated that the FCs adsorb and unfold efficiently upon whipping at equal concentrations of BLP obtained by CAE and UAAE. The concentration effect is indicative of the principles of foam physics [107]. An increase in protein content led to an increase in interfacial coverage and film thickness [108]. The stable FS demonstrated adequate interfacial elasticity and network formation in both extracts, a finding that is consistent with the results of recent studies on the foaming properties of plant proteins from legumes [109], [103]. Consequently, BLP obtained by CAE and UAAE demonstrated robust functionality, with consistently high levels of both FC and FS. These results indicate that BLP exhibited superior foaming properties in comparison to other reported leaf systems when evaluated on a like-for-like basis, while also preserving structural stability over time.

**Table 4:** Functional, physicochemical properties and in vitro digestibility for beetroot leaf protein after conventional alkaline extraction and ultrasound-assisted alkaline extraction.

Attributes	Concentration [%]	CAE	UAAE
Functional properties			
Foaming stability [%]	10 [%]	96.67 ± 4.71^b^	98.33 ± 2.36^b^
	15 [%]	94.03 ± 2.22^a^	94.10 ± 1.98^a^
Foaming capacity [%]	10 [%]	130.67 ± 0.0^a^	133.00 ± 0.0^a^
	15 [%]	148.33 ± 3.30^b^	148.33 ± 3.30^b^
Physicochemical properties			
Particle size [nm]		486.70 ± 21.15^a^	492.30 ± 35.0^a^
Zeta-potential [mV]		−24.03 ± 0.98^a^	–32.73 ± 2.35^b^
Interfacial tension [mN/m]		22.23 ± 2.96^a^	26.30 ± 3.67^b^
*In vitro* digestion			
*In vitro* protein digestibility [%]		66.22 ± 0.05^a^	67.07 ± 0.44^a^

Note: Results are shown as mean ± SEM of three replicates. Significant differences (p < 0.05) within the same column are indicated by different letters (a, b).

### 3.6 Physical properties

#### 3.6.1 Particle size and zeta potential

The distribution of surface charge has been demonstrated to have a significant impact on interfacial behaviour, including phenomena such as foaming and emulsification [110]. As demonstrated in Table 4, a statistically significant divergence in the Zeta-potential values was observed between the extracts. It was found that BLP after CAE was −24.03 ± 0.98 mV, and BLP obtained by UAAE was –32.73 ± 2.35 mV, indicating a significant difference among the groups, as expected. The greater negative charge exhibited by BLP after UAAE suggested a more pronounced electrostatic repulsion among protein particles when compared to BLP obtained by UAAE, which generally supported dispersion and thereby reduced the tendency to form aggregates. These shifts have been shown to be compatible with cavitation-driven changes in surface chemistry, including exposure/reorientation of charged amino-acid residues and modifications in adsorbed low-molecular-weight components Li et al. [111]. Furthermore, it has been demonstrated that greater absolute surface charge is often accompanied by greater solubility and reduced aggregation in plant proteins [112]. Similar increases in negative charge after ultrasound have been reported for chickpea isolates Kang et al. [113], soybean glycinin [114] and sunflower meal proteins [110]. On the other hand, hydrodynamic particle size exhibited no significant difference, as illustrated in Table 4. This outcome suggested that, under the present conditions, functionality gains are driven more by surface charge than by size reduction. Therefore, it can be concluded that UAAE enhanced the electrostatic stabilisation of the protein dispersions without measurably altering the average aggregate size. This interpretation is consistent with reports that ultrasound can enhance dispersion stability primarily by increasing the Zeta potential and modifying inter-particle interactions [39]. However, it should be emphasised that particle size changes depend strongly on factors such as concentration, pH, ionic strength and the composition of the surrounding matrix [115]. Moreover, a more negative potential is observed to support enhanced aqueous solubility and more persistent interfacial films, a finding that is consistent with the beneficial foaming and emulsion behaviour observed for BLP in this study.

#### 3.6.2 Interfacial tension

A comprehensive understanding of interfacial behaviour is instrumental in elucidating the reasons behind the enhanced stability of certain emulsions and foams in comparison to others [116]. As demonstrated in Table 4, the BLP after CAE exhibited a lower oil–water interfacial tension (22.23 mN/m) in comparison to the BLP obtained by UAAE (26.3 mN/m). This finding indicated that lower values are generally consistent with droplet disruption during homogenisation and are frequently associated with enhanced initial foam and emulsion formation [117]. Nevertheless, protein interfacial tension is time dependent and susceptible to measurement artefacts (sedimentation or slow adsorption kinetics in pendant drop tests), thereby requiring careful interpretation of absolute values [118], [119]. Furthermore, it is important to consider the reasons why BLP after CAE may result in a lower interfacial tension. It has been demonstrated in previous research on faba bean that the presence of soluble, rapidly adsorbing protein fractions can result in a more pronounced depression of air–water or oil–water tension over shorter periods of time [120], [121]. In contrast, BLP obtained by UAAE exhibited a more significant negative potential of –32.7 mV, as compared to −24.0 mV for BLP after CAE, as determined in the present study. This suggested that electrostatic repulsion between droplets or particles is enhanced, leading to improved long-term stability, even with interfacial tensions that are not among the lowest [122]. A review of the existing literature on plant-protein emulsifiers revealed a consistent conclusion that interfacial tension is not the only factor that determines stability [123]. It was determined that interfacial rheology (film elasticity/viscoelasticity), surface charge, and adsorption dynamics are significantly contributing factors, if not more so, during storage [124]. It can be concluded that the lower interfacial tension exhibited by BLP obtained by CAE facilitated the formation step (easier droplet break-up), whilst the more negative charge of BLP after UAAE led to enhanced stability (slower flocculation/creaming) through stronger electrostatic repulsion and potentially more cohesive interfacial films. These observations are consistent with those made after the ultrasound processing of other plant proteins [120]. Recent studies have reported similar trends, with mixed legume proteins designed for emulsification demonstrating that the composition and adsorption rate can influence tension and stability [125]. Zein-based systems have highlighted the importance of interfacial rheology during storage, and comprehensive reviews have emphasised that interfacial tension reduction, interfacial elasticity, and charge must be considered together to ensure accurate predictions of functionality [126], [90].

### 3.7 SDS-Page

**Fig. 7:**
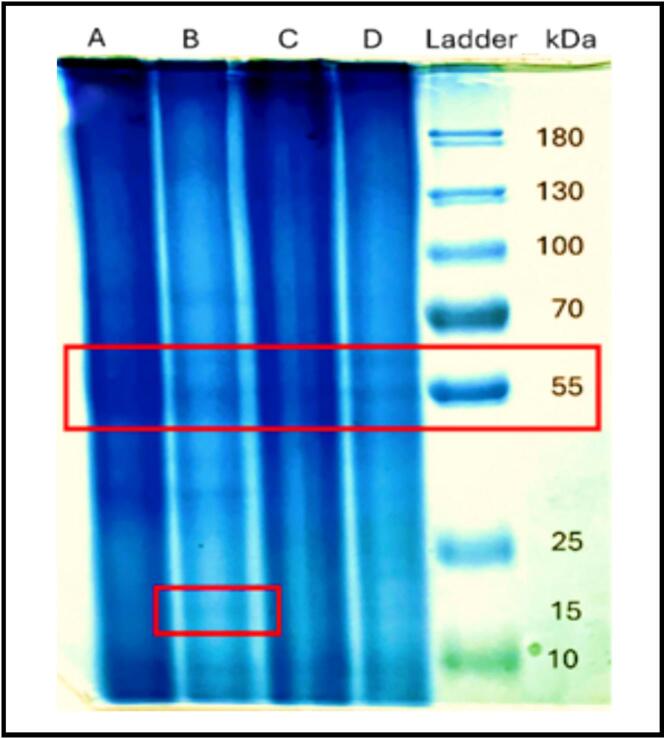
SDS-Page of BLP obtained by conventional alkaline extraction and ultrasound-assisted alkaline extraction at different concentrations: A: 10 mg BLP after CAE, B: 5 mg BLP after CAE, C: 10 mg BLP after UAAE, D: 5 mg BLP after UAAE.

SDS-PAGE was utilised to profile the protein fractions in BLP after CAE and UAAE. Across all loadings, the gels were dominated by low-molecular-weight bands (10–70 kDa), with a strong band at 55 kDa and a weaker band at 14–15 kDa (Fig. 7). These sizes corresponded to the large (RbcL, 55 kDa) and small (RbcS, 14–15 kDa) subunits of Rubisco, the major soluble protein in green leaves. This finding was consistent with previous reports on leaf-protein concentrates and isolates in SDS-PAGE, where the same doublet was also reported [14], [104]. Extraction studies, including those employing ultrasound-based methodologies, have confirmed the abundance of Rubisco in sugar-beet leaves and other foliage, thereby further validating this conclusion [127]. From a functional perspective, Rubisco-rich leaf proteins exhibited desirable interfacial and techno-functional characteristics (e.g., emulsifying/foaming), which are in alignment with our previous findings and with recent studies on Brassicaceae leaf concentrates [128], [129]. A similar low-MW pattern has been described for broccoli, cabbage and cauliflower leaf proteins, aligning with earlier observations for green-leaf proteins [27]. Furthermore, the presence of diffuse bands in Fig. 7, as distinguished from the expected sharpness, may be attributed to various plant-specific effects. These included the presence of residual phenolics/pigments, which have been observed to interfere with the binding and migration of SDS [130], [131]. Additional causes may include the presence of salts or an overload of reagents, as well as the under-resolution of small proteins on standard gels.

### 3.8 Amino acid composition

**Fig. 8:**
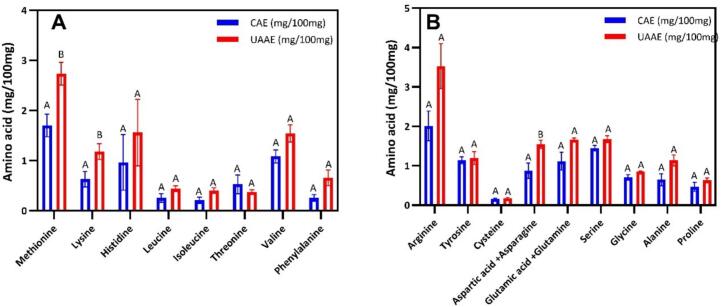
Comparison of essential (A) and non-essential (B) amino acid contents in BLP after CAE (blue) and BLP after UAAE (red) expressed as mg/100 mg dry weight. Data are presented as mean ± standard deviation (n = 3). Different letters above bars indicate statistically significant differences (p < 0.05).

Fig. 8 provided a comparison of the essential and non-essential amino acid (AA) profiles of BLP obtained by CAE versus UAAE. The present study demonstrated that the BLP obtained by UAAE exhibited higher concentrations of several EAAs, most significantly methionine (2.733 mg/100 mg) and lysine (1.182 mg/100 mg) in comparison to the BLP obtained by CAE (1.702 mg/100 mg and 0.631 mg/100 mg, respectively). Increases were also observed for histidine, leucine, isoleucine, threonine, valine, and phenylalanine, while tyrosine and cysteine showed no statistical difference. Furthermore, the sum of EAAs across the two samples revealed a significant difference, with BLP after UAAE reaching 8.891 mg/100 mg compared with 5.628 mg/100 mg for BLP obtained by CAE, indicating a considerably more abundant essential AA profile. For non-EAAs, aspartic acid/asparagine increased significantly with BLP after UAAE (1.547 mg/100 mg vs. 0.871 mg/100 mg), and the total non-EAA pool increased from 9.131 mg/100 mg to 13.415 mg/100 mg. These shifts are consistent with the ability of ultrasound to improve extraction and selectively enrich soluble protein fractions, often of the Rubisco composition, in leaf systems, as indicated by the findings of this study. This suggested that the shifts do not result from chemical formation of new amino acids. Similar EAA gained after ultrasound-assisted alkali extraction have been reported for pea protein isolates, where UAAE improved extraction and functional attributes alongside compositional changes [132]. This study suggested that acoustic cavitation may transiently unfold proteins and enhance mass transfer, which can expose hydrophobic and aromatic residues (Trp/Tyr/Phe), reduce large aggregates, and increase recovery of well-solubilising proteins. These effects modify the measurable AA profile of the recovered isolate. Recent mechanistic and review papers confirmed that ultrasound-induced exposure of hydrophobic/fluorescent residues and rearrangement of non-covalent interactions in plant proteins have been observed, aligning with the pattern demonstrated in this study [133], [53]. In sugar-beet leaves specifically, UAAE has been shown to enhance total protein yield and enrich Rubisco relative to high-voltage electric discharge, thereby supporting the hypothesis that ultrasound enhances the proportion of nutritionally valuable, soluble proteins recovered from leaves [127]. These results also demonstrated the potential for wider comparisons amongst legumes and seed meals, with ultrasound-assisted extraction from chickpea and pistachio meal exhibiting higher solubility, changes in hydrophobic amino acid exposure, and enhanced functionality, with composition and process parameters (power, time, temperature, pH) determining the degree of change [113], [93]. In addition, a comparative analysis of cavitation technologies (ultrasonic bath (bath (USB), ultrasonic plate (US-plate), ultrasonic probe (US-probe)) for pea protein further confirmed that enhanced cavitation improved protein recovery rate and modulated compositional results [134]. Further studies on canola and soy by-products have demonstrated similar findings in terms of protein recovery under UAAE, thereby reinforcing the validity of the mechanism observed [135]. Consequently, the enhanced EAA profile and elevated non-EAA levels observed in the BLP obtained by UAAE aligned with the existing literature, which demonstrated the efficacy of ultrasound-assisted alkaline extraction in enhancing yield, enriching nutritionally desirable fractions, and optimising functional properties. This suggested that BLP after by UAAE might be considered a promising ingredient for functional foods, supplements, and sustainable protein applications.

### 3.9 *In vitro* digestion

*In vitro* protein digestibility (IVPD) has been shown to provide a practical indicator of the bioavailability of novel proteins by simulating key steps of human gastrointestinal digestion [7]. As demonstrated in Table 4, under the same digestion protocol, BLP exhibited comparable IVPD after UAAE (67.06%) and CAE (66.20%), with no significant difference observed. These values were significantly higher than those reported for beet-leaf concentrates prepared by alkaline extraction at pH 10 (IVPD ≈ 40.76%) [27]. The observed difference in results with Sedlar et al. [27] is likely indicative of variations in process conditions. The pH of the extraction process and the accompanying washing steps have been shown to influence protein solubility, aggregation tendency, and the co-extraction of polyphenols and other antinutritional factors (ANFs) [52]. These factors can modify enzyme accessibility during the digestive process. The structural data from secondary structure of BLP, which included reduced β-sheet proportion under UAAE, tighter but more accessible networks, and higher negative zeta-potential, is consistent with greater protease accessibility [136]. This greater protease activity helped to explain the elevated IVPD, even if BLP obtained by UAAE and CAE showed similar results in this study [137], [138]. Furthermore, the mechanism of ultrasound cavitation has been demonstrated to facilitate the partial unfolding of proteins and the reduction of ANFs (phenolic-protein complexes, trypsin inhibitors) [4]. This process has been shown to enhance the accessibility of enzymes, thereby contributing to the enhancement of biological activity. These findings were supported by the higher IVPD observed in this study, in comparison to several green-leaf, including olive-leaf proteins [15], mulberry leaf proteins [41], and Brassicaceae leaves (cabbage, cauliflower, broccoli) [27], which typically reported lower digestibility under comparable in-vitro conditions. Consequently, the findings suggested that beetroot-leaf proteins, predominantly soluble Rubisco, are highly digestible upon effective extraction and purification from inhibitory co-extracts. Furthermore, it was observed that UAAE exhibited no detrimental impact on digestibility when compared to CAE.

## 4 Conclusion

In summary, response surface methodology was employed to optimise ultrasound-assisted alkaline extraction (UAAE) of beetroot-leaf protein (BLP) at pH 11, 40 min, 27.2 °C. This approach resulted in a higher extraction yield (9.0% vs 5.5%) and protein content (70.81% vs 57.42%) than conventional alkaline extraction (CAE). Across the range of characterisation methods, UAAE produced a protein that was both better-ordered and more accessible: The FTIR results indicated the preservation of the backbone structure, accompanied by a shift in the secondary structure elements towards increased β-sheet and decreased β-turn/α-helix regions. The XRD analysis revealed an enhancement in crystallinity, with a percentage of 50.46% compared to 28.09% in the control sample. Furthermore, the SEM/AFM analysis demonstrated a reduction in the surface roughness of the lamellae and the presence of a more interconnected globular network. In terms of electrokinetic properties, BLP obtained by UAAE exhibited a more negative Zeta potential of –32.73 mV, in comparison to BLP after CAE −24.03 mV. Additionally, BLP obtained by CAE demonstrated a lower oil–water interfacial tension of 22.23 mV, as compared to 26.3 mV for BLP after UAAE, suggesting a dual functionality in emulsion formation (CAE) and long-term stability (UAAE). While extraction at a pH of 11 enhances solubility and yield, it can also induce denaturation, deamidation, and oxidation, affecting functional and nutritional properties and posing industrial challenges linked to neutralisation costs, equipment durability, and wastewater management. The present work provides a relevant and significant finding that will assist industry in the design of milder, more sustainable extraction strategies that will preserve protein functionality. The composition of the samples was analysed, revealing an increase in essential and non-essential amino acids (methionine and lysine in particular), with no change to thermal stability (TGA/DSC) and preservation of Rubisco subunits (55 and 14–15 kDa) by SDS-PAGE. The process of creaming was driven by protein concentration and temperature-insensitive over a period of seven days (4 °C and 25 °C). It was observed that foam capacity demonstrated a positive correlation with the concentration of BLP, and that foam stability remained high at 10–15%. Furthermore, it was determined that BLP obtained by UAAE exhibited a lighter colour and a gel-like texture, which was beneficial to the creation of a clean-label structuring. The IVPD was found to be elevated for both extracts, with a range of 66–67%, thereby exceeding several reported green-leaf references. Consequently, the UAAE offered a convenient, cost-effective, and efficient approach that enhanced yield, structure, and composition without compromising digestibility. This finding positioned beetroot-leaf protein as a promising sustainable ingredient and facilitated the valorisation of the beetroot-leaf into functional food ingredient.

## CRediT authorship contribution statement

**El Mehdi Raoui:** Writing – original draft, Visualization, Validation, Investigation, Formal analysis. **Sofia Gruber:** Writing – original draft, Visualization, Validation, Investigation, Formal analysis. **Milad Hadidi:** Writing – review & editing, Visualization, Supervision, Investigation, Formal analysis, Conceptualization. **Wisnu Arifan Anditya Sudjarwo:** Writing – review & editing, Methodology, Formal analysis. **Alexander Einschütz Lopez:** Writing – review & editing, Methodology, Formal analysis. **Jose L. Toca-herrera:** Writing – review & editing, Methodology. **Christian Leopold Lengauer:** Writing – review & editing, Methodology. **Marc Pignitter:** Writing – review & editing, Supervision, Funding acquisition, Conceptualization.

## Declaration of competing interest

The authors declare that they have no known competing financial interests or personal relationships that could have appeared to influence the work reported in this paper.
