# Supplementary material for: Unveiling the potential of beetroot leaf as a sustainable source of proteins: insights into ultrasound-assisted extraction, functional properties and in vitro digestibility
Source: Ultrason Sonochem. 2026 Jan 24;126:107751. doi: 10.1016/j.ultsonch.2026.107751 (PMC12887188; doi:10.1016/j.ultsonch.2026.107751)
Supplement: Supplementary Data 1 [file mmc1.docx]

**Unveiling the potential of beetroot leaf as a sustainable source of proteins: Insights into ultrasound-assisted extraction, functional properties and *in vitro* digestibility**

El Mehdi RAOUI^a,b^, Sofia GRUBER^a^, Milad HADIDI^a*^, Wisnu Arifan Anditya SUDJARWO^c^, Alexander EINSCHÜTZ LOPEZ^c^, Jose L. TOCA-HERRERA^c^, Christian Leopold LENGAUER^d^, Marc PIGNITTER^a*^

^a^ Institute of Physiological Chemistry, Faculty of Chemistry, University of Vienna, 1090 Vienna, Austria

email: [raoui.el.mehdi@univie.ac.at](mailto:raoui.el.mehdi@univie.ac.at), [milad.hadidi@univie.ac.at](mailto:milad.hadidi@univie.ac.at), [marc.pignitter@univie.ac.at](mailto:marc.pignitter@univie.ac.at)

^b^ Vienna Doctoral School in Chemistry (DoSChem), University of Vienna, Vienna, Austria

email: [raoui.el.mehdi@univie.ac.at](mailto:raoui.el.mehdi@univie.ac.at)

^c^ Institute of Biophysics, Department of Bionanosciences (DBNS), University of Natural Resources and Life Sciences (BOKU), 1190 Vienna, Austria

email: [alexander.el@boku.ac.at](mailto:alexander.el@boku.ac.at), [jose.toca-herrera@boku.ac.at](mailto:jose.toca-herrera@boku.ac.at)

^d^Institute of Mineralogy and Crystallography, Faculty of Earth Sciences, University of Vienna, 1090 Vienna, Austria

email: [christian.lengauer@univie.ac.at](mailto:christian.lengauer@univie.ac.at)

*Corresponding authors: Marc Pignitter, Milad Hadidi, email: [marc.pignitter@univie.ac.at](mailto:marc.pignitter@univie.ac.at); [milad.hadidi@gmail.com](mailto:milad.hadidi@gmail.com)

Table S1: HPLC gradient separation program for amino acid determination

| Time (min) | Flow Rate | Eluent A (%) | Eluent B (%) | Eluent C (%) |
| --- | --- | --- | --- | --- |
| Initial | 0.4 | 100 | 0 | 0 |
| 0.5 | 0.4 | 99 | 1 | 0 |
| 18 | 0.4 | 95 | 5 | 0 |
| 19 | 0.4 | 91 | 9 | 0 |
| 28 | 0.4 | 83 | 17 | 0 |
| 35 | 0.4 | 0 | 60 | 40 |
| 38 | 0.4 | 100 | 0 | 0 |
